# Association of Adiponectin and Vitamin D With Tumor Infiltrating Lymphocytes and Survival in Stage III Colon Cancer

**DOI:** 10.1093/jncics/pkab070

**Published:** 2021-07-23

**Authors:** Frank A Sinicrope, Qian Shi, Thomas C Smyrk, Richard M Goldberg, Steven J Cohen, Sharlene Gill, Morton S Kahlenberg, Suresh Nair, Anthony F Shield, Balkrishna N Jahagirdar, Sawyer B Jacobson, Nathan R Foster, Michael N Pollak, Steven R Alberts

**Affiliations:** 1 Division of Oncology and Mayo Clinic Comprehensive Cancer Center, Rochester, MN, USA; 2 Alliance Statistics and Data Center, Mayo Clinic, Rochester, MN, USA; 3 West Virginia University Cancer Institute, Morgantown, WV, USA; 4 Fox Chase Cancer Center, Philadelphia, PA, USA; 5 British Columbia Cancer Agency, Vancouver Cancer Centre, Vancouver, BC, Canada; 6 Surgical Oncology Associates of South Texas, San Antonio, TX, USA; 7 Lehigh Valley Hospital, Allentown, PA, USA; 8 Wayne State University, Karmanos Cancer Institute, Detroit, MI, USA; 9 Metro Minnesota Community Oncology Research Consortium, Saint Paul, MN, USA; 10 McGill University, Montreal, QC, Canada

## Abstract

**Background:**

Adipocyte-derived adiponectin may play a role in the host inflammatory response to cancer. We examined the association of plasma adiponectin with the density of tumor-infiltrating lymphocytes (TILs) in colon cancers and with vitamin D, clinicopathological features, and patient survival.

**Methods:**

Plasma adiponectin and 25-hydroxyvitamin D [25(OH)D] were analyzed by radioimmunoassay in 600 patients with stage III colon cancer who received FOLFOX-based adjuvant chemotherapy (NCCTG N0147 [Alliance]). TIL densities were determined in histopathological sections. Associations with disease-free survival (DFS), time to recurrence, and overall survival were evaluated by multivariable Cox regression adjusting for potential confounders (ie, body mass index, race, TILs, and N stage). All statistical tests were 2-sided.

**Results:**

We found a statistically significant reduction in adiponectin, but not 25(OH)D, levels in tumors with high vs low TIL densities (median* *=* *6845 vs 8984 ng/mL; *P* = .04). A statistically significant reduction in adiponectin was also observed in obese (body mass index >30 kg/m^2^) vs nonobese patients (median* *=* *6608 vs 12 351 ng/mL; *P* < .001), in men vs women (median* *=* *8185 vs 11 567 ng/mL; *P* < .001), in Blacks vs Whites or Asians (median* *=* *6412 vs 8847 vs 7858 ng/mL; *P* < .03), and in those with fewer lymph node metastases (N1 vs N2: median* *=* *7768 vs 9253 ng/mL; *P* = .01). Insufficiency of 25(OH)D (<30 ng/mL) was detected in 291 (48.5%) patients. In multivariable analyses, neither adiponectin nor 25(OH)D were associated with a statistically significant difference in DFS, overall survival , or time to recurrence in models adjusted for potential confounders. We found a statistically significant association of TILs with prognosis, yet no such interaction was observed for the association of adiponectin with TILs for DFS.

**Conclusions:**

Lower circulating adiponectin levels were associated with a statistically significant increase in TIL densities in colon cancers, indicating an enhanced antitumor immune response. In contrast to TILs, neither adiponectin nor 25(OH)D was independently prognostic.

Colorectal cancer (CRC) is the third most common cancer and is second only to lung cancer as a cause of cancer-related mortality in the United States (1). Obesity is an established risk factor in this malignancy, and it is postulated that adiponectin may mediate the biological link between obesity and CRC (2). Although the mechanism for this potential link is unknown, adipocyte-derived adiponectin may play a role in immune regulation and in the host inflammatory response to cancer (3,4). Adiponectin, the most abundant hormone secreted by adipose tissue, senses metabolic stress and modulates metabolic adaption by targeting the innate immune system under physiological and pathological conditions (5). Epidemiological studies suggest an inverse association between plasma adiponectin levels and risk of developing CRC (6,7). This inverse relationship was observed among men, but not women, in the Nurses’ Health Study and Health Professionals Follow-up Study (6). In a meta-analysis of patients with established CRC, a statistically significant decrease in adiponectin levels was observed compared with non CRC controls (8). Limited data suggest an association between prediagnostic plasma adiponectin and risk of CRC-specific and overall mortality (9). However, the only prospective study of adiponectin measured at the time of diagnosis of CRC was not prognostic (10), and postdiagnosis data are lacking.

Plasma adiponectin was found to be associated with vitamin D levels in non-CRC patients (11). Vitamin D insufficiency (<30 ng/mL) is relatively common in healthy individuals and in CRC populations (12). Serum 25-hydroxyvitamin D [25(OH)D] was inversely associated with CRC risk (13), and some studies suggest that 25(OH)D insufficiency in patients with established CRC may be associated with worse clinical outcome (14-16). Individuals deficient in vitamin D had statistically significantly higher levels of the serum inflammatory biomarkers interleukin-6 (IL-6) and C-reactive protein (17). Interestingly, a prior study found that a high 25(OH)D level was associated with a lower risk of developing CRC with an intense immune reaction (18), suggesting that vitamin D may influence the tumor-host interaction. In a study of the 25(OH)D score measured post-CRC diagnosis, its association with CRC-specific mortality differed by the extent of peritumoral lymphocytic reaction (19).

To date, adiponectin has not been analyzed in relationship to the tumor immune microenvironment. We sought to test the hypothesis that adiponectin may play a role in the host inflammatory response to colon cancer, indicated by tumor-infiltrating lymphocytes (TILs). In patients with CRC, studies indicate that TILs can independently predict recurrence and survival (20-22). However, only limited data exist for adiponectin or vitamin D in patients with established CRC, and their impact on survival is largely unknown. We determined the association of postsurgical plasma adiponectin levels with TIL densities, 25(OH)D, clinicopathological features, and clinical outcome in patients with stage III colon cancers from a phase III adjuvant trial of FOLFOX-based chemotherapy (NCCTG N0147 [Alliance]) (23).

## Methods

### Study Population

Among 2686 patients with resected stage III colon cancer who had been randomly assigned to adjuvant FOLFOX alone or combined with cetuximab, we randomly selected 600 patients (300 per arm) for analysis of adiponectin and 25(OH)D (NCCTG N0147; ClinicalTrials.gov Identifier: NCT00079274). Right-sided tumors were defined as located proximal to the splenic flexure. N1 tumors had 1-3 metastatic regional lymph nodes; N2 tumors had at least 4 nodes. All patients provided written informed consent at the time of clinical trial enrollment. All data analyses shown here were approved by Mayo Clinic Institution Review Board.

### Adiponectin and Vitamin D Assays

Plasma adiponectin and 25(OH)D were analyzed in processed and stored blood obtained at adjuvant study registration. Biomarker concentrations were measured by radioimmunoassay (laboratory of M. Pollak, McGill University, Montreal, QC, Canada) as previously described (24). Circulating levels of total and high molecular weight adiponectin were measured in duplicate using standard enzyme-linked immunosorbent assay methods. Because total and high molecular weight adiponectin levels were almost perfectly correlated (Spearman *P *=* *.99), we report total adiponectin. 25(OH)D was extracted from serum or plasma with acetonitrile, and samples were then assayed using an equilibrium radioimmunoassay procedure using an antibody specific for 25(OH)D (24). Blinded quality control (QC) samples were included with test samples, and pooled QC specimens were added to each of the batches. For both assays, masked QC samples were interspersed among case samples. All laboratory personnel were blinded to patient outcomes. The mean coefficient of variation of the assay was 8%.

### Histologic Examination of TILs

A representative hematoxylin and eosin-stained tumor section from each patient was scanned at low power to identify areas with the most intraepithelial TILs. Once identified, 5 consecutive 40X fields were counted, and mean TILs per high power field (HPF) were calculated by dividing the total number of TILs by 5. All cases were scored independently by 2 gastrointestinal pathologists blinded to clinical and molecular data (25). Cutoffs for TIL densities were previously determined in N0147 tumors based on their association with patient (disease-free survival [DFS]) and categorized as low (≤3 per HPF) vs high (>3 per HPF) TILs (25).

### DNA Mismatch Repair (MMR) Status and *KRAS*/*BRAF* Mutation Analysis

Prospectively collected tumor tissues were analyzed for MMR status by analysis of MLH1, MSH2, and MSH6 proteins using immunohistochemistry. Tumors with deficient MMR were defined as having absent expression of 1 or more MMR protein. Testing for a *BRAF* (c.1799T>A V600E) mutation in exon 15 and *KRAS* mutations in codons 12 and 13 of exon 2 was previously described (26).

### Statistical Analysis

Adiponectin and 25(OH)D were analyzed as continuous variables in the primary analysis. As binary variables, adiponectin was dichotomized at the median, and 25(OH)D was dichotomized at 30 ng/ml with lower levels regarded as insufficiency (12). The association of adiponectin with TILs was prespecified as the primary analysis and was analyzed by the Kruskal Wallis test. Associations between adiponectin and 25(OH)D and with clinicopathological features were assessed by Fisher exact, Pearson χ^2^, *t* test, and Kruskal-Wallis tests as appropriate. The association between adiponectin and clinical outcomes were as follows: DFS, time from randomization to recurrence or death from any cause; overall survival (OS), time from randomization to death from all causes; and time to recurrence (TTR) as time from randomization to recurrence. Distributions of DFS, OS, and TTR were estimated by the Kaplan-Meier method. Biomarkers were analyzed in relationship to clinical outcome as continuous variables using a relative risk model with cubic splines and as binary variables in multivariable Cox regression. Multivariable models included variables that were considered to be potential confounders. Interaction tests were performed. A 2-sided *P* value of less than  .05 was considered statistically significant and was not adjusted for multiple comparisons. SAS software version 9.4 and R version 3.6.2 were used (SAS Institute, Cary, NC).

## Results

### Association of Plasma Adiponectin and 25(OH)D With Patient Characteristics and TILs

**Figure 1.**
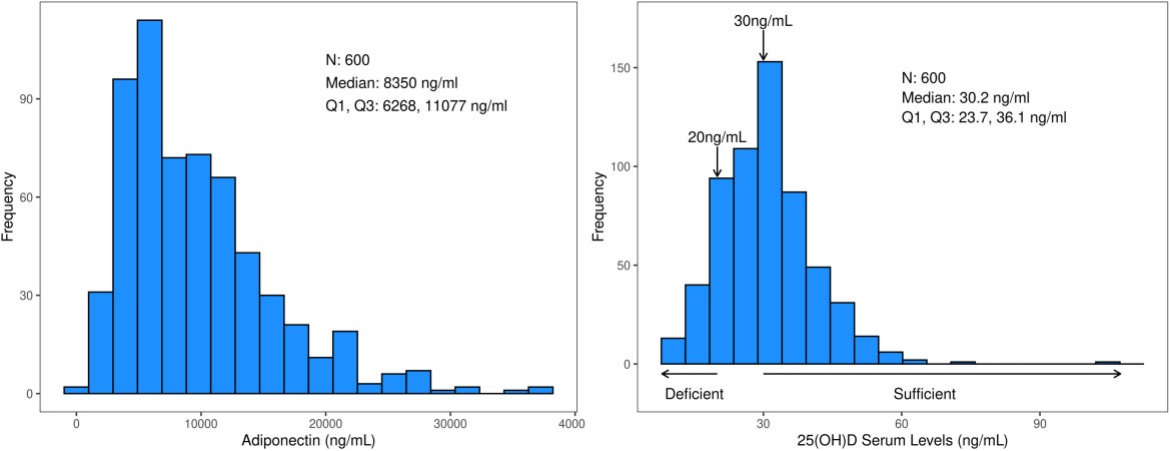
Frequency distributions of postsurgical plasma adiponectin (**left**) and 25-hydroxyvitamin D (**right**) in 600 patients with surgically resected stage III colon cancers treated with adjuvant FOLFOX-based chemotherapy.

The frequency distribution and median levels of plasma adiponectin and 25(OH)D are shown in Figure 1. We found a statistically significant decrease in the level of adiponectin in patients whose tumors had high vs low TIL densities (median* *=* *6845 vs 8984 ng/mL; *P* = .04) (Table 1). Furthermore, a statistically significant and inverse association between adiponectin level and body mass index (BMI) category was observed whereby obese patients had a lower median adiponectin level (median* *=* *6608 ng/mL) than did those of normal weight (median* *=* *10 693 ng/mL) or underweight (median* *=* *18 010 ng/mL; *P* < .001). A statistically significant decrease in adiponectin was observed in men vs women (median* *=* *8185 vs 11 567 ng/mL; *P *<* *.001), in those aged 65 years or younger (median* *=* *7663 vs 9279 ng/mL; *P *=* *.007), and in Blacks vs Whites or Asians (median* *=* *6412 vs 8847 vs 7858 ng/mL; *P* < .03) (Table 1). Findings by race were not explained by differences in BMI, which was similar by race. Adiponectin levels differed by N stage in that a lower level was associated with fewer regional lymph node metastases (N1 vs N2: median* *=* *7768 vs 9253 ng/mL; *P* = .01). Adiponectin was not associated with a statistically significant correlation with 25(OH)D (Spearman *r *=* *−.0038; *P* = .93) or with T stage, DNA MMR, or the mutational status of *KRAS* or *BRAF* genes.

**Table 1.** Association between plasma adiponectin level and clinicopathological variables

Variable	No.	Plasma adiponectin level, ng/mL			*P* ^a^
		Mean (SD)	Median	Q 1, Q3	
Age, y					.007
≤65	420	9372.2 (5877.4)	7663.0	5106.0, 12 419.5	
>65	180	10 751.7 (6382.7)	9279.0	6268.8, 13 581.8	
Race					.002
Asian	28	9503.2 (5247.7)	7858.0	5110.2, 13 653.0	
Black	53	7245.5 (4791.3)	6412.0	4086.0, 8988.0	
White	502	10 065.3 (6115.2)	8847.0	5472.5, 12 842.8	
Sex					<.001
Female	284	11 567.9 (6528.8)	10 439.0	6420.2, 14 978.5	
Male	316	8184.7 (5111.8)	6816.5	4466.2, 10 694.8	
BMI					<.001
Underweight	10	16944.1 (9775.4)	18009.5	8890.0, 25 879.0	
Normal	159	12 218.2 (6908.1)	10693.0	6906.0, 15 800.0	
Overweight	210	9454.6 (5508.4)	8350.0	52 38.5, 12 040.8	
Obese	218	7938.8 (4759.6)	6608.0	45 96.0, 10 348.0	
T stage					.48
T1 or T2	96	9348.5 (6397.1)	6961.0	5084.0, 12 162.8	
T3	441	9896.8 (6013.4)	8580.0	5328.0, 12 729.0	
T4	63	9677.5 (5933.8)	8741.0	5411.5, 12 695.5	
N stage					.01
N1	365	9372.3 (6008.2)	7768.0	4959.0, 12 069.0	
N2	235	10 428.7 (6099.8)	9253.0	5733.0, 13 262.0	
Performance status					.93
0	453	9791.9 (6015.2)	8435.0	5308.0, 12 729.0	
1	141	9706.5 (6178.2)	8145.0	5215.0, 11 977.0	
2	6	11 212.0 (7671.0)	10 068.0	4993.0, 17 817.5	
Tumor location					.74
Left	286	9822.6 (5996.2)	8390.0	5331.2, 13 020.8	
Right	303	9721.2 (5957.8)	8354.0	5250.0, 12 285.5	
TILs					.04
Low (≤3)	248	10 163.4 (5881.1)	8983.5	5575.5, 13 467.0	
High (>3)	100	9294.3 (6612.9)	6844.5	4945.5, 11 389.8	
*BRAF*					.85
Mutant	198	9548.7 (5729.9)	8147.0	5501.8, 12 046.0	
Wild type	357	9829.8 (6070.0)	8434.0	5215.0, 12 729.0	
*KRAS*					.61
Mutant	73	10 287.8 (6416.9)	8492.0	5443.0, 12 729.0	
Wild type	482	9645.0 (5875.3)	8350.0	5278.5, 12 552.5	
MMR					.96
dMMR	73	9784.9 (5995.4)	8580.0	5443.0, 11 972.0	
pMMR	504	9762.8 (5955.6)	8303.0	5279.5, 12 687.5	

a Kruskal-Wallis rank sum test. All statistical tests were 2-sided. BMI = body mass index; dMMR = deficient mismatch repair; MMR = mismatch repair; pMMR = proficient mismatch repair; TILs = tumor-infiltrating lymphocytes; Q = Quartile.

A statistically significant reduction in plasma 25(OH)D, as a continuous variable, was found in Blacks vs Whites or Asians (*P *<* *.001) (Table 2). Differences by sex were statistically, but not clinically significant. A statistically significant decrease in 25(OH)D levels was found in the relatively few patients with poor performance status. Insufficiency of 25(OH)D (<0 ng/mL) was detected in 49% (291 of 600) of study participants. Consistent results were found for the dichotomous 25(OH)D by patient race (*P* < .001) and sex (*P* < .03) (Supplementary Table 1, available online). Specifically, insufficiency of 25(OH)D was more common in Blacks vs Whites or Asians (73.6% vs 45% vs 53.6%) and in women vs men (53.2% vs 44.3%), with both achieving statistical significance. 25(OH)D was not statistically significantly associated with BMI or adiponectin level. Neither the association of the continuous nor of the dichotomous 25(OH)D level with TIL density achieved statistical significance (Table 2; Supplementary Table 1, available online).

**Table 2.** Association between continuous vitamin D level and clinicopathological variables

Variable	No.	Vitamin D level, ng/mL			*P* ^a^
		Mean (SD)	Median	Q1, Q3	
Age, y					.74
≤65	420	30.9 (10.2)	30.3	24.0, 35.9	
>65	180	30.7 (10.3)	30.0	23.2, 36.5	
Race					<.001
Asian	28	30.7 (9.9)	29.6	25.0, 35.7	
Black	53	25.2 (8.7)	23.1	18.9, 30.3	
White	502	31.5 (10.3)	31.0	24.2, 36.7	
Sex					.03
Female	284	30.0 (10.9)	29.2	22.7, 35.7	
Male	316	31.5 (9.5)	30.8	25.6, 36.4	
BMI					.09
Underweight	10	30.7 (8.8)	32.4	26.2, 35.6	
Normal	159	31.8 (10.2)	30.6	24.9, 36.6	
Overweight	210	31.3 (9.9)	31.5	23.4, 37.5	
Obese	218	29.6 (10.5)	29.3	23.1, 34.0	
T stage					.51
T1 or T2	96	31.9 (10.9)	31.0	24.6, 38.3	
T3	441	30.7 (10.1)	30.2	23.5, 35.9	
T4	63	29.9 (9.3)	29.8	23.5, 34.5	
N stage					.60
1-3	365	31.1 (11.0)	30.2	23.7, 36.6	
4	235	30.3 (8.8)	30.2	23.7, 35.2	
Performance status					.047
0	453	31.3 (10.4)	30.8	24.2, 36.7	
1	141	29.3 (9.5)	29.2	22.7, 34.9	
2	6	26.0 (4.5)	25.8	22.7, 28.1	
Tumor location					.34
Left	286	31.4 (10.8)	30.7	24.2, 36.9	
Right	303	30.4 (9.6)	29.8	23.6, 35.7	
TILs					.07
Low (≤3)	248	31.4 (10.7)	30.2	24.2, 37.6	
High (>3)	100	29.1 (9.4)	29.2	22.6, 34.2	
*BRAF*					.82
Mutant	198	31.4 (11.5)	30.1	23.7, 36.5	
Wild type	357	30.5 (9.6)	30.3	23.4, 36.0	
*KRAS*					.47
Mutant	73	29.8 (10.1)	30.2	22.5, 36.1	
Wild type	482	31.0 (10.3)	30.2	23.7, 36.1	
MMR					.90
dMMR	73	30.6 (10.2)	31.2	22.7, 36.1	
pMMR	504	30.8 (10.3)	30.1	23.7, 35.9	

a Kruskal-Wallis rank sum test. All statistical tests were 2-sided. BMI = body mass index; dMMR = deficient mismatch repair; MMR = mismatch repair; pMMR = proficient mismatch repair; TILs = tumor-infiltrating lymphocytes; Q = Quartile.

### Association of Plasma Adiponectin and 25(OH)D Levels With Patient Clinical Outcome

**Figure 2.**
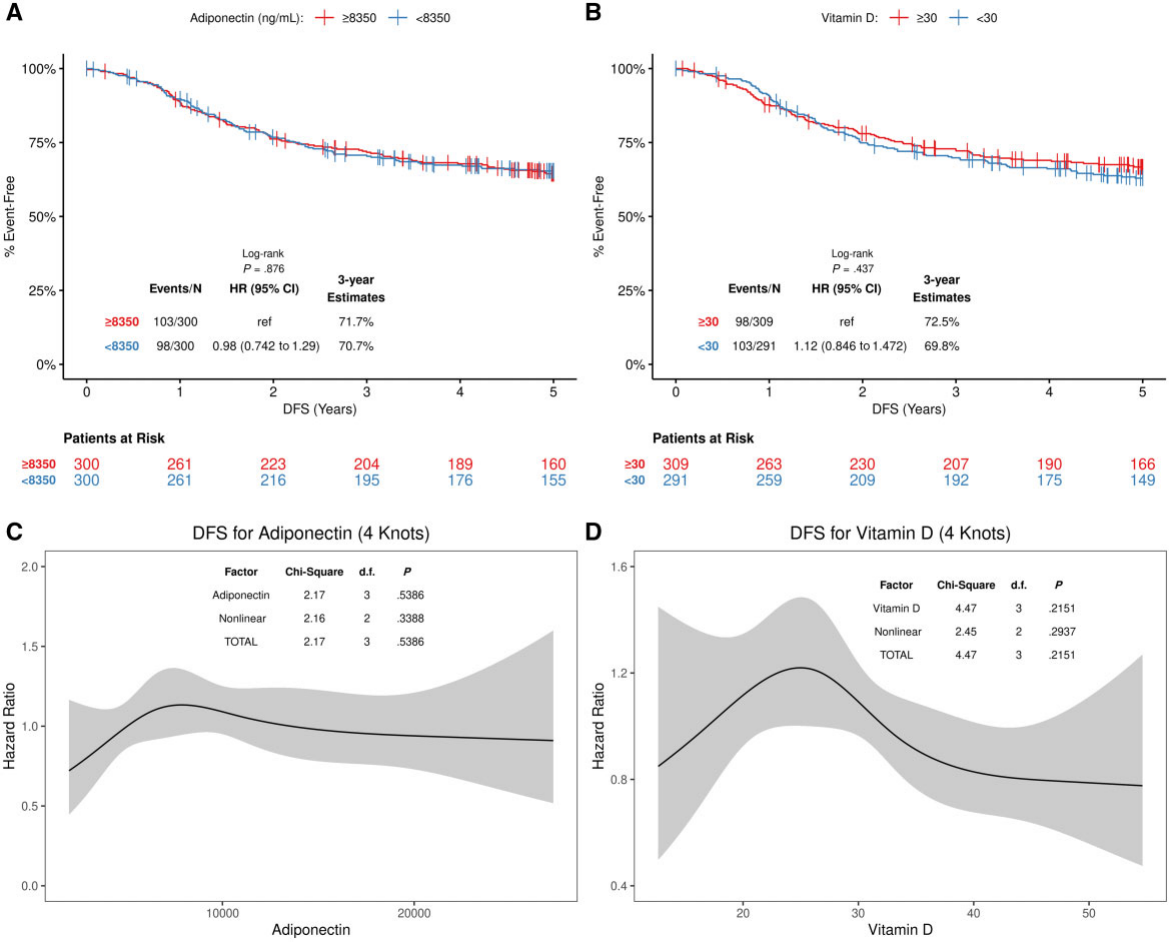
Univariate association of postsurgical plasma adiponectin and 25(OH)D with DFS in patients with stage III colon cancer. Variables are analyzed as dichotomous using Kaplan-Meier plots (**A, B**) or as continuous using a relative risk model with cubic splines (**C, D**). Adiponectin level was dichotomized at the median value (**A**), and 25(OH)D level was dichotomized as insufficient (<30 ng/mL) vs sufficient (>30 ng/mL) (**B**). All statistical tests were 2-sided. 25(OH)D = 25-hydroxyvitamin D; CI = confidence interval; d.f. = degrees of freedom; DFS = disease-free survival; HR = hazard ratio.

Univariately, plasma adiponectin was not statistically significantly associated with DFS as a dichotomous variable (cut at median; hazard ratio [HR]* *=* *0.98, 95% confidence interval [CI]* *=* *0.74 to 1.29; *P* = .88) shown in a Kaplan Meier plot (Figure 2, A) or a continuous variable shown by cubic spline (Figure 2, C). No association was found between adiponectin and OS (HR = 0.90, 95% CI* *=* *0.66 to 1.22; *P* = .49) or TTR (HR = 1.03, 95% CI* *=* *0.75 to 1.34; *P *=* *1.00) (data not shown). We then constructed a multivariable model that included adiponectin and potential confounding variables based on observed associations (Table 1) and the literature. The association of adiponectin with patient DFS did not achieve statistical significance as a continuous (adjusted HR [HR_adj_]* *=* *1.01, 95% CI* *=* *0.98 to 1.04; adjusted *P* [*P*_adj_] = .57) or a dichotomous (data not shown) variable in a multivariable model adjusted for treatment arm, race, BMI, TILs, and N stage (Table 3). In this model, statistically significantly poorer DFS was observed for tumors with low vs high TILs (HR_adj_ = 1.77, 95% CI* *=* *1.11 to 2.81; *P*_adj_ = .02) and N2 vs N1 stage (HR_adj_ = 2.08, 95% CI* *=* *1.44 to 2.99; *P*_adj_ < .001) (Table 3). Adiponectin was not statistically significantly associated with OS or TTR as either a continuous or dichotomous variable.

**Table 3.** Multivariable analysis of plasma adiponectin and patient disease-free survival

Variable	Events/Total	HR (95% CI)	*P*
Adiponectin (step size: 1000)		1.01 (0.98 to 1.04)	.57^a^
Treatment arm			
FOLFOX	56/169	Referent	
FOLFOX + cetuximab	66/169	1.09 (0.76 to 1.58)	.63^a^
Race			.34^b^
Asian	3/14	0.47 (0.15 to 1.51)	.20^a^
Black	9/22	1.09 (0.55 to 2.18)	.81^a^
White	110/302	Referent	
BMI			.41^b^
Normal	28/88	Referent	
Obese	54/133	1.50 (0.93 to 2.41)	.10^a^
Overweight	38/112	1.26 (0.77 to 2.07)	.36^a^
Underweight	2/5	1.02 (0.23 to 4.49)	.97^a^
TILs			
Low (≤3)	99/241	1.77 (1.11 to 2.81)	.02^a^
High (>3)	23/97	Referent	
N Stage			
N1	59/214	Referent	
N2	63/124	2.08 (1.44 to 2.99)	<.001^a^

a Covariate Wald *P* value. All tests were 2-sided. BMI = body mass index; CI = confidence interval; HR = hazard ratio; TILs = tumor-infiltrating lymphocytes.

b Type 3 likelihood-ratio *P* value. All tests were 2-sided.

Univariately, insufficient vs sufficient 25(OH)D was not statistically significantly associated with DFS (HR = 1.12, 95% CI* *=* *0.85 to 1.47; *P* = .44) (Figure 2, B), OS (HR = 1.20, 95% CI* *=* *0.88 to 1.63; *P* = .25), or TTR (HR = 1.11, 95% CI* *=* *0.83 to 1.49; *P* = .47). Similarly, the continuous 25(OH)D was not statistically significantly associated with DFS (Figure 2, D) or with other outcome variables. In contrast, low vs high TIL density was associated with poorer patient DFS (HR = 1.89, 95% CI* *=* *1.20 to 2.94; *P* = .005) that achieved statistical significance with 3-year survival rates of 65.4% vs 80.5%, respectively. In a multivariable model that included potential confounders, 25(OH)D as a continuous variable was not associated with patient DFS (HR_adj_* *=* *1.00, 95% CI* *=* *0.98 to 1.02; *P*_adj_ = 1.00) (Table 4) or with OS or TTR (data not shown). In this model, TILs (low vs high: HR = 1.77, 95% CI* *=* *1.11 to 2.83; *P* = .02) and N stage (HR = 2.10, 95% CI* *=* *1.46 to 3.02; *P *<* *.001) were statistically significantly associated with DFS (Table 4). We also examined 25(OH)D as a dichotomous variable whereby insufficiency (<30 ng/mL) was not associated with a statistically significant difference in DFS, OS, or TTR (data not shown). Furthermore, we dichotomized 25(OH)D according to the World Health Organization definition of vitamin D deficiency (<20 ng/mL) and found that it was again not statistically significantly prognostic for DFS (data not shown).

**Table 4.** Multivariate analysis of plasma 25-hydroxyvitamin D and patient disease-free survival

Variable	Events/Total	HR (95% CI)	*P*
Vitamin D ng/mL (step size: 1)		1.00 (0.98 to 1.02)	1.00^a^
Treatment arm			
FOLFOX	56/169	Referent	
FOLFOX + cetuximab	66/169	1.11 (0.77 to 1.59)	.58^a^
Race			.32^b^
Asian	3/14	0.46 (0.14 to 1.46)	.19^a^
Black	9/22	1.07 (0.53 to 2.16)	.85^a^
White	110/302	Referent	
BMI			.46^b^
Normal	28/88	Referent	
Obese	54/133	1.45 (0.91 to 2.30)	.12^a^
Overweight	38/112	1.24 (0.76 to 2.03)	.39^a^
Underweight	2/5	1.13 (0.27 to 4.78)	.87^a^
TILs			
Low (≤3)	99/241	1.77 (1.11 to 2.83)	.02^a^
High (>3)	23/97	Referent	
N Stage			
N1	59/214	Referent	
N2	63/124	2.10 (1.46 to 3.02)	<.001^a^

a Covariate Wald *P* value. All tests were 2-sided. BMI = body mass index; CI = confidence interval; HR = hazard ratio; TILs = tumor-infiltrating lymphocytes.

b Type 3 likelihood-ratio *P* value. All tests were 2-sided.

We constructed 3-way interaction models that examined the association of adiponectin and 25(OH)D (adjusting for race and BMI) by TILs and N stage subgroups. No statistically significant relationships were found indicating that the observed associations were not interdependent. Of note, TIL density did not differ by BMI category (*P *=* *.50) (data not shown). Furthermore and given observed differences in adiponectin and 25(OH)D levels by patient sex (Tables 1 and 2), analysis of 25(OH)D with clinical outcome variables (TTR, DFS, OS) by sex or TIL density failed to reveal any statistically significant associations after adjustment for covariates.

## Discussion

We analyzed adiponectin and 25(OH)D in postsurgical blood samples from patients with stage III colon cancer treated in a phase III trial of adjuvant chemotherapy. Based on preclinical data suggesting that adiponectin may influence the host inflammatory response to cancer, we determined the association of adiponectin with TIL density in the tumor immune microenvironment. Lower adiponectin levels were associated with a statistically significant increase in TIL density, indicating an inverse relationship between adiponectin and antitumor immunity. This result is consistent with data in a murine model where adiponectin deficiency was associated with tumors with an increased inflammatory infiltrate compared with tumors in nonadiponectin-deficient mice (27). Adiponectin has been shown to reduce T-cell and B-cell recruitment, induce production of anti-inflammatory cytokines such as IL-10 and an inhibitor of metalloproteinase-1, and inhibit pro-inflammatory chemokines such as IL-6 and TNF-α (5,28). Furthermore, adiponectin was shown to impede inflammation-induced tumorigenesis (29). In contrast to adiponectin, we did not find a statistically significant association between the level of plasma 25(OH)D and TIL density. In another report, higher plasma 25(OH)D levels were associated with a lower risk of CRCs with a high density of CD3^+^ T cells (18). We found that lower adiponectin levels were statistically significantly associated with cancers showing fewer regional lymph node metastases (N1 vs N2 stage). Relevant to this finding is our prior observation of statistically significantly higher TIL densities in N1 vs N2 stage colon cancers (*P *=* *.0001) (25), yet no interaction was found between TIL densities, N stage, and adiponectin in the current study.

Importantly, we confirmed the reported inverse relationship of adiponectin level to BMI in our dataset (30,31). A statistically significant and inverse relationship between adiponectin and BMI category was observed, with the lowest adiponectin levels found among obese patients. Although adiponectin is exclusively secreted by adipocytes, its paradoxical reduction in obesity may reflect reduced secretion from visceral fat rather than subcutaneous fat. Hypertrophic adipocytes synthesize monocyte chemotactic protein-1 leading to infiltration of macrophages in white adipose tissue accompanied by elevated local TNF-α and increased free fatty acid concentrations that suppress adiponectin secretion (32). Other factors may include dysregulation of the adiponectin gene (*ADIPOQ*) in obesity (33).

We observed differences in adiponectin by sex whereby men had statistically significantly lower levels compared with women. This observation may be explained by higher serum androgens in men (34), and the finding of increased adiponectin levels in older vs younger men may be due to an age-related decline in testosterone levels. In this regard, adiponectin levels were shown to decrease after testosterone administration in hypogonadal men (35). In women, adiponectin has been shown to decrease with the transition to menopause (36). We found statistically significantly lower adiponectin levels among Blacks compared with Whites or Asians, which was not explained by differences in BMI by race. However, lower adiponectin levels among Blacks vs Whites was attributed to adiponectin’s association with BMI in the population-based race-ethnic Northern Manhattan Study (37). Differences in adiponectin levels may also have a hereditary component, and further analysis of single nucleotide polymorphisms in adiponectin-related genes may provide further insight (38).

Whereas plasma adiponectin was associated with vitamin D levels in nonCRC patients (11), no such relationship was observed in our CRC cohort. We observed that nearly one-half of patients were vitamin D insufficient (<30 ng/mL), which far exceeds the 8% prevalence of vitamin D insufficiency in the US general population (39). We found a higher rate of vitamin D insufficiency among Black vs White or Asian. In addition to dietary factors and sunlight exposure, race can influence vitamin D status in that melanin in skin reduces the rate of vitamin D biosynthesis (12,40). Furthermore, racial differences in the prevalence of common genetic polymorphisms can influence vitamin D levels; for example, Blacks are more likely to have the T allele at rs7041 and less likely to have the A allele at rs4588 vs Whites (41). Differences by sex for the continuous vitamin D variable were modest; however, vitamin D insufficiency was statistically significantly more common in women vs men, which is consistent with an increased prevalence of hypovitaminosis D in women especially after menopause (42).

Despite the inverse association of adiponectin levels and TIL densities that we observed, adiponectin was not statistically significantly associated with patient DFS, OS, or TTR univariately or after adjustment for potential confounders. To our knowledge, these are the first data for postsurgical adiponectin and clinical outcome in CRC patients. Adiponectin at diagnosis of CRC was also not prognostic in a prospective study in 344 consecutive cases (10). Regarding prediagnostic adiponectin, 2 large observational cohorts found a statistically significant and independent association of higher levels with reduced CRC-specific and OS that was more evident in patients with metastatic disease (9). A potential explanation may be an elevation of adiponectin during weight loss, which is a known predictor of poor prognosis in cancer patients. Adiponectin is an insulin-sensitizing hormone, and low levels are associated with an increased risk of type 2 diabetes independent of other risk factors (43). Adiponectin mediates its insulin-sensitizing effect through activation of AMP-activated protein kinase (AMPK) and peroxisome proliferator-activated receptor–alpha pathways with resultant suppression of tumor growth in animal models (44). As with adiponectin, plasma 25(OH)D was not statistically significantly associated with clinical outcome variables in our cohort. However, a study of resected stage III colon cancer patients found that a higher predicted 25(OH)D score was associated with improved survival outcomes (16). Of note, 25(OH)D scores were calculated based on serial patient questionnaire data, but not measurements in blood samples. Another study of 1598 patients with stage I to III CRC found that patients with the highest vs lowest tertile of postoperative plasma 25(OH)D levels had lower CRC-specific and all-cause mortality that achieved statistical significance (45). 25(OH)D levels, however, were not prognostic in patients with stage IV CRC (n = 515) from a clinical trial cohort (46). We interpret our results and those in stage IV cancers to suggest that any effect of vitamin D on prognosis is attenuated or lost once the disease has metastasized either to regional lymph nodes (stage III) or to distant sites (stage IV). In contrast, a statistically significant association of TILs with prognosis was found in our cohort with low vs high TILs being associated with poorer patient survival as we previously reported (22,25).

Strengths of our study include same stage patients with uniform treatment in a clinical trial with long-term and meticulous follow-up data. Blood was collected from all patients prior to initiation of chemotherapy, such that the time period between biomarker sampling and initiation of adjuvant treatment was relatively constant. Whereas other studies have used cancer prediagnosis measurements (47), our study is the first to examine postdiagnostic plasma adiponectin levels in relation to survival outcomes in CRC patients. Study limitations include retrospective biomarker analysis that was not prespecified and the single assay timepoint. Importantly, measurement of 25(OH)D at a single timepoint was shown to provide an accurate assessment of an individual’s long-term vitamin D status (48). Similarly, adiponectin levels were shown to remain stable over time (49). We cannot exclude the possibility that the modest sample size of our study may have contributed to the failure to detect a prognostic effect for either biomarker.

In conclusion, we identified a novel inverse association of adiponectin with the intratumoral antitumor immune response indicated by TIL density. Validation of this association in an independent cohort of patients with CRC is warranted. In contrast to TILs, neither adiponectin nor 25(OH)D was found to be prognostic.

## Funding

Research reported in this publication was supported by the National Cancer Institute (NCI) of the National Institutes of Health (NIH) under award numbers U10CA180821, U10CA180882; U10CA180863, CCSRI 021039 (CCTG); U10CA180868 (NRG); U10CA180888, and UG1CA233163 (SWOG). NCCTG N0147 received funds from Sanofi Aventis. The study was also supported by NCI R01CA210509 (to FAS).

## Notes

**Role of the funders:** The funders had no role in the design of the study or in the data collection, analysis, or interpretation. Furthermore, the funders did not participate in the writing of the manuscript or in the decision to submit the manuscript for publication.

**Disclosures:** FAS is a co-inventor of intellectual property with Roche Ventana Medical Systems (Tucson, AZ) and may receive royalties paid to Mayo Clinic. No other relevant conflicts related to the subject matter of this manuscript are reported by the study authors.

**Author contributions:** Study conceptualization: FAS. Data curation: QS. Formal analysis: SBJ, NRF, QS. Funding acquisition: FAS, SRA. Investigation: TCS, RMG, SJC, SG, MSK, SN, AFS, BNJ, MNP, SRA. Methodology: MNP. Project administration/supervision: FAS. Writing: original draft: FAS. Writing-review and editing: all authors.

## Data Availability

Data from published Alliance or North Central Cancer Treatment Group (NCCTG) trials can be accessed by submission of an Alliance Data Sharing Request Form (concepts@allianceNCTN.org). Requests to utilize biomarker data published in this manuscript should also be directed to the corresponding author.

## Supplementary Material

pkab070_Supplementary_Data
