# Supplementary material for: Association of Adiponectin and Vitamin D With Tumor Infiltrating Lymphocytes and Survival in Stage III Colon Cancer
Source: JNCI Cancer Spectr. 2021 Jul 23;5(5):pkab070. doi: 10.1093/jncics/pkab070 (PMC8410141; doi:10.1093/jncics/pkab070)
Supplement: pkab070_Supplementary_Data [file pkab070_supplementary_data.pdf]

Supplementary Table 1. Association between plasma 25(OH)D level and clinicopathological variables

Variable	Plasma 25(OH)D level Sufficient (≥ 30 ng/ml) No. (%)	Plasma 25(OH)D level Insufficient (< 30 ng/ml) No. (%)	Total No.	P^a
Total No.	309	291	600	
Age, y				0.63
≤ 65	219 (52.1)	201 (47.9)	420	
> 65	90 (50.0)	90 (50.0)	180	
Race				<0.001
Asian	13 (46.4)	15 (53.6)	28	
Black	14 (26.4)	39 (73.6)	53	
White	276 (55.0)	226 (45.0)	502	
Gender				0.03
Female	133 (46.8)	151 (53.2)	284	
Male	176 (55.7)	140 (44.3)	316	
BMI				0.53
Underweight	6 (60.0)	4 (40.0)	10	
Normal	83 (52.2)	76 (47.8)	159	
Overweight	114 (54.3)	96 (45.7)	210	
Obese	104 (47.7)	114 (52.3)	218	
T Stage				0.81
T1 or T2	52 (54.2)	44 (45.8)	96	
T3	226 (51.2)	215 (48.8)	441	
T4	31 (49.2)	32 (50.8)	63	
N Stage				0.74
N1	186 (51.0)	179 (49.0)	365	
N2	123 (52.3)	112 (47.7)	235	
Performance status				0.11[b]
0	241 (53.2)	212 (46.8)	453	
1	67 (47.5)	74 (52.5)	141	
2	1 (16.7)	5 (83.3)	6	
Location				0.19
Left	156 (54.5)	130 (45.5)	286	
Right	149 (49.2)	154 (50.8)	303	
TILs				0.40
Low (≤3)	129 (52.0)	119 (48.0)	248	
High (>3)	47 (47.0)	53 (53.0)	100	
BRAF				0.72
Mutant	100 (50.5)	98 (49.5)	198	
Wild-type	186 (52.1)	171 (47.9)	357	
KRAS				0.88
Mutant	37 (50.7)	36 (49.3)	73	
Wild-type	249 (51.7)	233 (48.3)	482	
MMR				0.72
dMMR	39 (53.4)	34 (46.6)	73	

pMMR	258 (51.2%)	246 (48.8%)	504	
Adiponectin				0.22
< 8350 (median)	162 (54.0%)	138 (46.0%)	300	
≥ 8350	147 (49.0%)	153 (51.0%)	300	

[a]Pearson's Chi-squared test. All statistical tests were 2-sided. BMI = body mass index; MMR = mismatch repair; dMMR = deficient mismatch repair; pMMR = proficient mismatch repair; TILs = tumor infiltrating lymphocytes

[b]Fisher's Exact Test
